# Evaluation of the therapeutic value of conventional transpedal lymphography for the treatment of inguinal lymphatic fistulas after lymphadenectomy

**DOI:** 10.1186/s42155-026-00713-8

**Published:** 2026-06-12

**Authors:** Christian Wolfram, Thomas J. Vogl, Katrin Eichler, Tatjana Gruber-Rouh

**Affiliations:** Department of Radiology and Nuclear Medicine, Johann Wolfgang Goethe-University Hospital Frankfurt, Building 23C, Basement, Theodor-Stern-Kai 7, 60590 Frankfurt am Main, Germany

**Keywords:** Lymphangiography, Lymphatic intervention, Lipiodol, Postoperative lymphatic leakage, Inguinal lymphatic fistula

## Abstract

**Purpose:**

To evaluate therapeutic effectiveness and safety of lipiodol-based conservative transpedal lymphangiography in sealing persistent inguinal lymphatic fistulas after lymphadenectomy and to determine whether therapeutic success is influenced by the amount of injected Lipiodol or the volume of lymphatic drainage.

**Materials and methods:**

From January 2003 to June 2023, 184 patients underwent lymphangiography. Of these, 35 patients (24 males, 11 females; aged 24 to 87 years) met inclusion criteria (age ≥ 18 years, persistent lymphatic leakage after inguinal lymphadenectomy (> 3 weeks), following unsuccessful conservative management) and were subsequently included for statistical analysis. Lipiodol lymphangiography was performed via transpedal lymphatic vessel cannulation. Data collected included the following: age, sex, underlying disease, drainage volume before lymphangiography, Lipiodol amount, procedural details, time to fistula closure, imaging follow-ups, technical success, therapeutic success and its correlation with the volume of lymphatic leakage and the volume of the applied iodised oil, complications, and additional interventions. Statistical analysis utilised the Wilcoxon–Mann–Whitney and logistic regression tests (significance at *p* ≤ 0.05).

**Results:**

Therapeutic success was achieved in 22 patients (62.86%) without complications, with a mean resolution time of 7.13 days. Thirteen patients (37.14%) required additional interventions. No significant correlation was found between therapeutic success and either the amount of Lipiodol used (*p* = 0.51 and *p* = 0.49) or drainage volume (*p* = 0.82 and *p* = 0.79).

**Conclusion:**

Lipiodol-based lymphangiography is an effective and safe treatment for inguinal lymphatic fistulas. The lack of association of therapeutic outcome with Lipiodol or drainage volume appears to be more related to anatomical and disease-specific factors, suggesting individualised patient assessment is warranted.

## Background

Iodised oil-based conventional lymphangiography is a diagnostic imaging technique that enables the detailed visualisation of the lymphatic system, facilitating the evaluation of conditions such as lymphatic fistulas, lymphovenous malformations, and lymphatic obstructions through the injection of an oily contrast agent [1–7]. Lipiodol, an iodised oil derived from poppy seeds (Papaver somniferum var), transitioned from being solely a diagnostic contrast agent to a therapeutic tool by enabling the successful closure of lymphatic leaks [4, 5, 8–16]. Its mechanism of action involves the accumulation of Lipiodol at leak sites, which physically obstructs lymph flow and initiates a mild sterile inflammatory response that leads to fibrosis, ultimately occluding the leaking lymphatic vessels over time [17]. This dual capability—serving both diagnostic and therapeutic purposes—offers a distinct advantage over conventional imaging methods. Lastly, in those cases where lymphatic leakage cannot be managed through medical treatment, consisting of a low-fat, medium-chain triglyceride diet [18–20], somatostatin treatment [21, 22], or local therapies such as bed rest, elevation of the extremity and compression bandages, lymphatic drainage [23, 24], vacuum therapy [25–30], lymphangiography has proven to be a minimally invasive method for the treatment of lymphatic leakage. This contrasts with surgical treatments, which carry relatively high complication and mortality rates, reported at up to 38% and 25%, respectively, and do not guarantee complete closure of the fistula [10, 31–33].

The comparability of studies regarding the therapeutic effect of conventional lymphangiography is limited due to the analysis of different patient groups, on the one hand, and, on the other hand, the different volume ranges of lymphatic drainage. There is a notable lack of systematic research focused on homogeneous patient cohorts, especially concerning postoperative inguinal lymphatic fistulas.

To address this gap, the intention of the study was to evaluate therapeutic effectiveness and safety of lipiodol-based conservative lymphangiography in sealing persistent inguinal lymphatic fistulas after lymphadenectomy. The study also sought to determine whether there is a correlation between therapeutic success and the volume of injected Lipiodol, as well as the volume of lymphatic drainage.

## Methods

This is a retrospective observational study that was performed in one institution and approved by the local ethics committee. All procedures performed in studies involving human participants were in accordance with the ethical standards of the institutional and/or national research committee and with the 1964 Helsinki Declaration and its later amendments or comparable ethical standards. The requirement to obtain individual consent was waived.

### Patient population

**Fig. 1:**
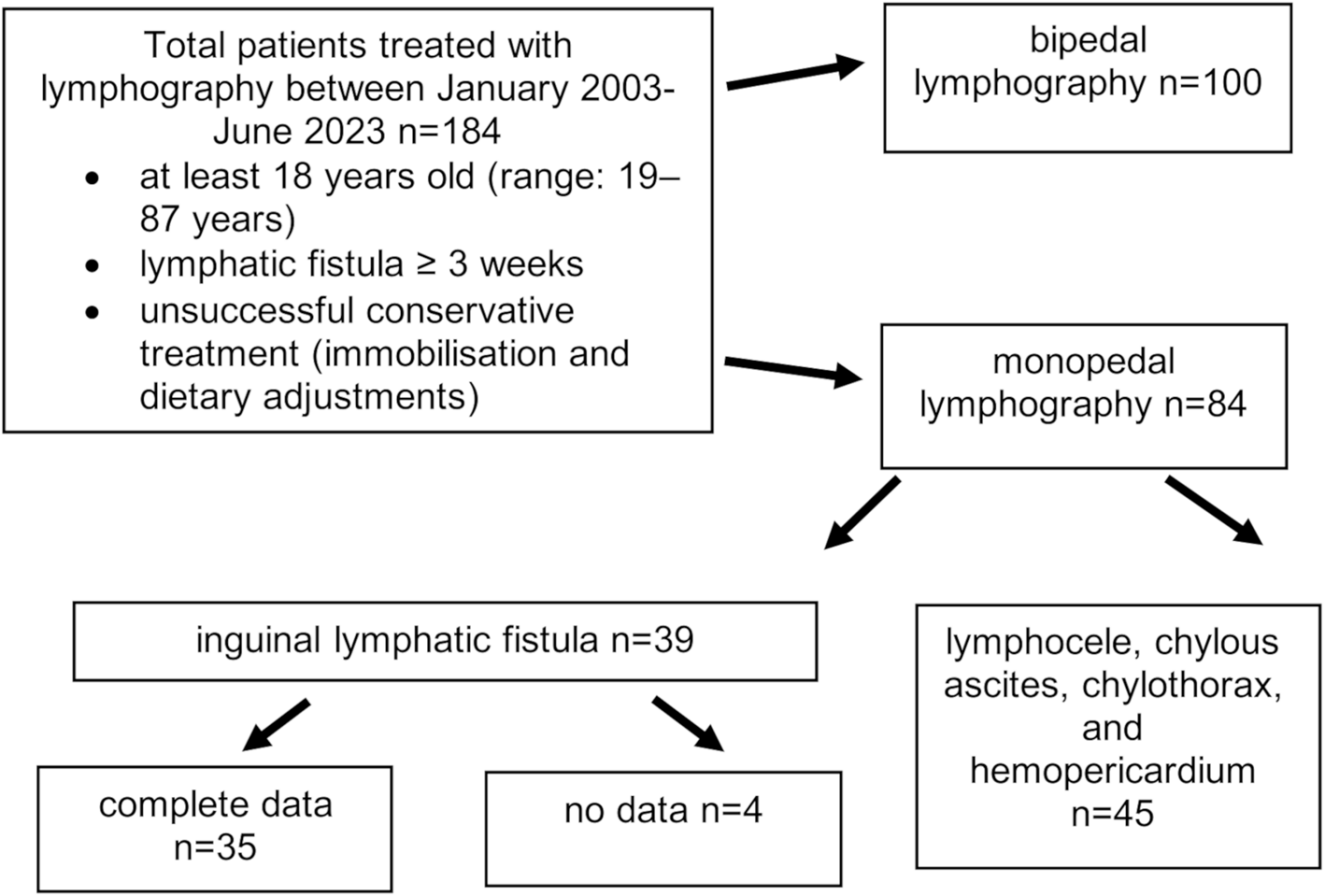
Flowchart of patient selection. Flow diagram illustrating the selection of patients treated with lymphangiography between January 2003 and June 2023 (*n* = 184). Inclusion criteria were age ≥ 18 years (range, 19–87 years), presence of a lymphatic fistula for at least 3 weeks, and unsuccessful conservative treatment (immobilisation and dietary adjustments). A distinction was made between monopedal and bipedal lymphangiography. Of the 184 patients, 84 were treated with monopedal lymphangiography. The diagnosis was categorised as inguinal lymph fistula, lymphocele, chylous ascites, chylothorax, and chylopericardium. Of these, 39 patients suffered from inguinal lymph fistulas. Exclusion criteria were incomplete medical records or the lack of a follow-up examination prior to the evaluation of the results. Only 4 were excluded from the statistical evaluation, while the other 35 patients underwent successful lymphangiography. This resulted in a sample size of 35 patients who were included in the final analysis

**Fig. 2:**
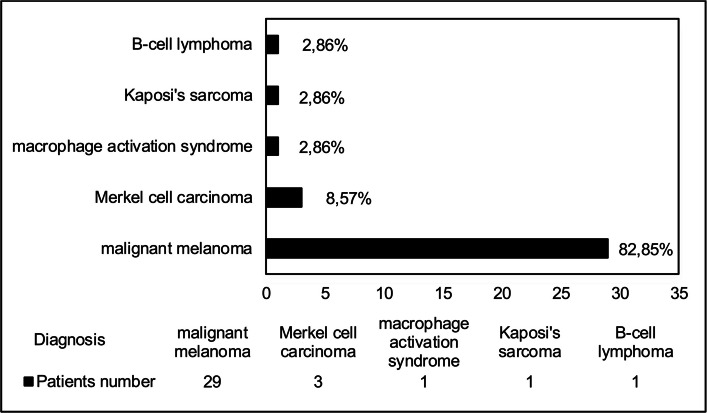
Overview of diagnoses causing inguinal lymphatic leakage. Bar chart displaying the underlying diagnoses in 35 patients with inguinal lymphatic leakage. Most lymphatic leakage cases in the study cohort were due to prior inguinal lymphadenectomy for cutaneous malignant melanoma (82.9%, *n* = 29), followed by Merkel cell carcinoma (8.6%, *n* = 3). Less common causes included macrophage activation syndrome (2.9%, *n* = 1), Kaposi’s sarcoma (2.9%, *n* = 1), and B-cell lymphoma (2.9%, *n* = 1)

Between January 2003 and June 2023, 184 patients were treated with lymphangiography in our department. Following the application of inclusion and exclusion criteria, 35 patients who developed lymphatic fistulas in the inguinal region were further recorded and analysed (Fig. 1). The details of the patients, including underlying diseases, age, sex, daily lymphatic drainage volume in ml before the lymphangiography procedure, the amount of Lipiodol administered, technical success, whether X-ray/CT performed after lymphangiography, clinical success, time to resolution, complications, and additional interventions are summarised in Table 1. The cohort included 11 females (31.4%) and 24 males (68.6%) patients, with a median age of 71 years. The age range was from 24 to 87 years, with a mean of 67.2 years. A significant factor contributing to lymphatic leakage was previous inguinal lymphadenectomy conducted for the diagnosis of cutaneous malignant melanoma, which accounted for 82.85% (*n* = 29) of the cases (Fig. 2).

**Table 1:** Patient characteristics and outcomes of lymphatic intervention

Patient no. and underlying disease	Age	Sex	Daily drain output (mL)	Amount of lipiodol administered (mL)	Technical success	X-ray/CT performed after lymphography	Clinical success	Time to resolution	Complication	Additional intervention
1/Macrophage activation syndrome	24	m	50	9	Yes	X-ray	No	Not available	No	Surgery
2/Cutaneous malignant melanoma	71	m	50	6	Yes	X-ray	No	Not available	No	Yes, further course is not available
3/Cutaneous malignant melanoma	43	w	100	8	Yes	X-ray	No	Not available	No	Yes, further course is not available
4/Cutaneous malignant melanoma	47	m	100	9	Yes	CT	Yes	2	No	No
5/Cutaneous malignant melanoma	66	w	100	9	Yes	X-ray	No	Not available	No	Yes, further course is not available
6/Cutaneous malignant melanoma	70	w	100	18	Yes	X-ray	Yes	2	No	No
7/Cutaneous malignant melanoma	77	m	100	9	Yes	X-ray	Yes	7	No	No
8/Cutaneous malignant melanoma	77	m	100	12	Yes	X-ray	No	Not available	No	Radiotherapy
9/Merkel cell carcinoma	80	w	100	8	Yes	X-ray	Yes	4	No	No
10/Cutaneous malignant melanoma	87	w	100	8	Yes	X-ray	Yes	7	No	No
11/Cutaneous malignant melanoma	57	m	120	9	Yes	X-ray	Yes	2	No	No
12/Cutaneous malignant melanoma	80	w	120	4	Yes	X-ray	Yes	2	No	No
13/B-cell lymphoma	51	m	125	8	Yes	X-ray	Yes	35	No	No
14/Cutaneous malignant melanoma	51	m	150	8	Yes	X-ray	No	Not available	No	Radiotherapy
15/Cutaneous malignant melanoma	75	m	150	9	Yes	X-ray	Yes	1	No	No
16/Cutaneous malignant melanoma	82	m	150	12	Yes	X-ray	Yes	11	No	No
17/Cutaneous malignant melanoma	68	m	170	10	Yes	X-ray	Yes	2	No	No
18/Cutaneous malignant melanoma	62	w	200	9	Yes	X-ray	Yes	2	No	No
19/Merkel cell carcinoma	79	m	200	9	Yes	X-ray	No	Not available	No	Surgery
20/Cutaneous malignant melanoma	81	m	200	9	Yes	X-ray	No	Not available	No	Radiotherapy
21/Cutaneous malignant melanoma	36	w	250	6	Yes	X-ray	Yes	18	No	No
22/Cutaneous malignant melanoma	66	m	250	9	Yes	X-ray	Yes	10	No	No
23/Cutaneous malignant melanoma	76	m	250	6	Yes	X-ray	Yes	2	No	No
24/Merkel cell carcinoma	78	m	250	8	Yes	X-ray	No	Not available	No	Yes, further course is not available
25/Cutaneous malignant melanoma	80	m	250	9	Yes	X-ray	Yes	6	No	No
26/Cutaneous malignant melanoma	80	w	250	9	Yes	X-ray	Yes	6	No	No
27/Kaposi ‘s sarcoma	47	m	300	9	Yes	X-ray	Yes	2	No	No
28/Cutaneous malignant melanoma	66	w	300	5	Yes	X-ray	Yes	2	No	No
29/Cutaneous malignant melanoma	80	m	325	9	Yes	X-ray	No	Not available	No	Radiotherapy
30/Cutaneous malignant melanoma	49	m	400	14	Yes	X-ray	Yes	2	No	No
31/Cutaneous malignant melanoma	68	m	400	8	Yes	X-ray	Yes	25	No	No
32/Cutaneous malignant melanoma	75	w	400	9	Yes	X-ray	No	Not available	No	Radiotherapy
33/Cutaneous malignant melanoma	70	m	500	10	Yes	X-ray	Yes	7	No	No
34/Cutaneous malignant melanoma	76	m	500	8	Yes	X-ray	No	Not available	No	Radiotherapy
35/Cutaneous malignant melanoma	77	m	500	5	Yes	X-ray	No	Not available	No	Surgery

### Lymphangiography procedure

**Fig. 3:**
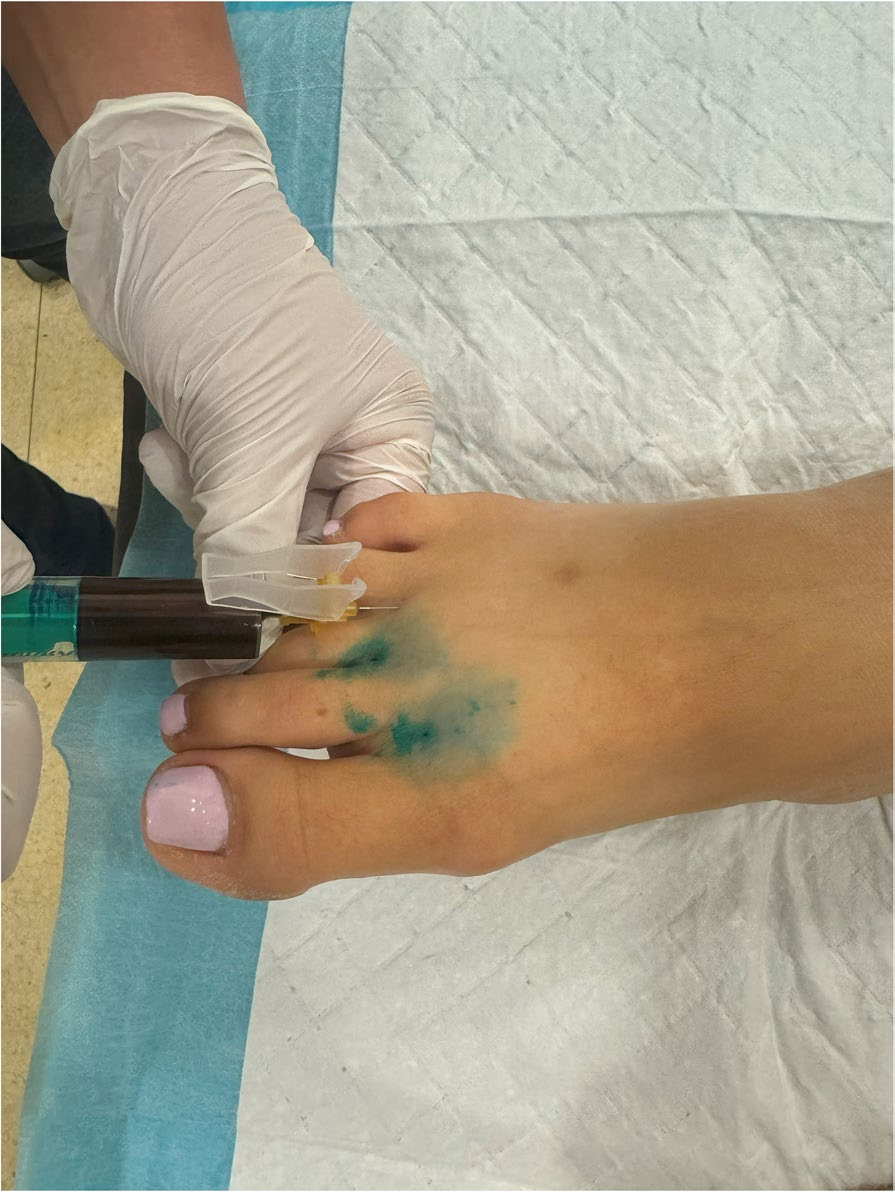
Subcutaneous injection of methylene blue in preparation for lymphangiography. A 42-year-old female patient with lymphatic fistula after inguinal lymphadenectomy. Documentation of the injection of mepivacaine hydrochloride methylene blue dye into the subcutaneous tissues of the first to third interdigital spaces

**Fig. 4:**
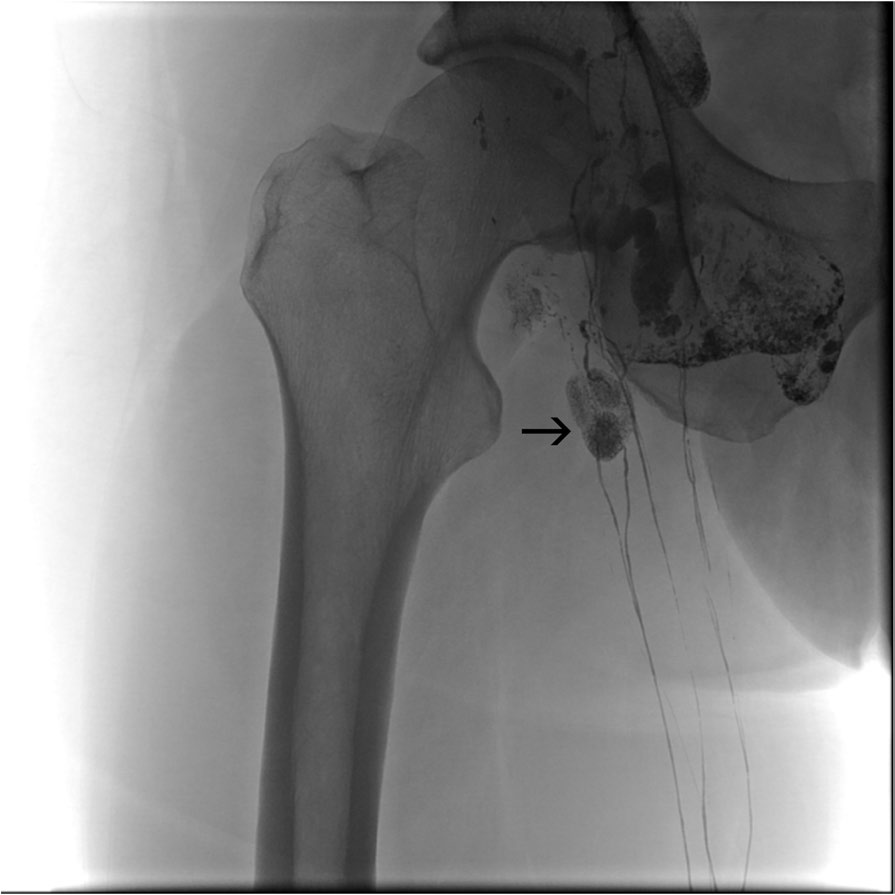
Tracking of contrast fluid flow via fluoroscopy. A 46-year-old male patient with lymphatic fistula after inguinal lymphadenectomy. Documentation of the accumulation of iodized oil in the region of the lymphatic fistula and flow into the drainage immediately after the lymphangiography

**Fig. 5:**
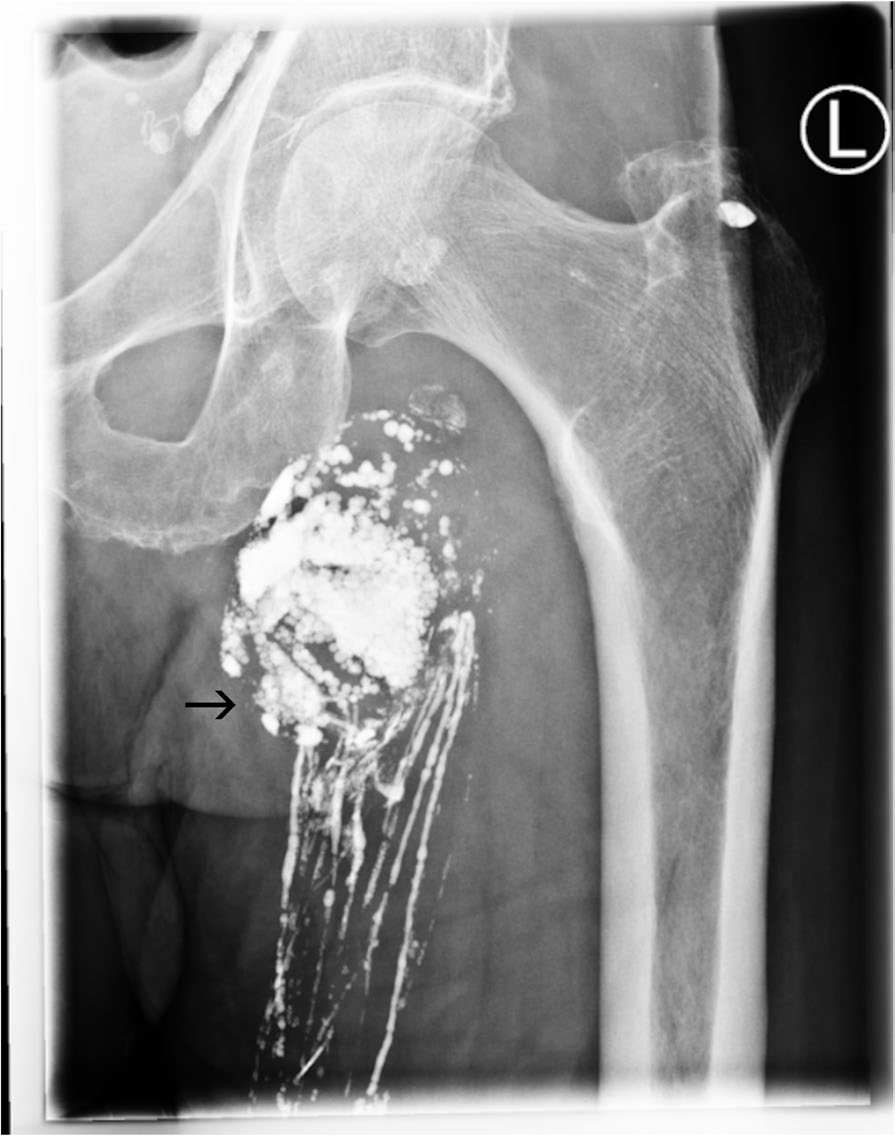
X-ray 24 h after intervention. An 82-year-old male patient with lymphatic fistula after inguinal lymphadenectomy. Documentation of the visualisation of the lymphatic fistula 24 h after the lymphangiography

All 35 patients underwent the same procedure. Following the disinfection of the feet (using Kodan®, Schülke and Mayr GmbH, Norderstedt, Germany), 2 ml of mepivacaine hydrochloride (Scandicaine® 1%, Astra Zeneca GmbH, Wedel, Germany) and 2 ml of methylene blue dye (Patent Blue V, Guerbet GmbH, Sulzbach, Germany) were injected into the subcutaneous tissues of the first to third interdigital spaces (Fig. 3). After the pedal lymphatic vessels had been adequately coloured, which took around 15 min, the patient was placed on an examination table of the X-ray radioscopic system Siemens Axiom Artis MP (Siemens, Forchheim, Germany). The foot was then disinfected again and covered with a sterile fenestrated drape. Following this step, a local anaesthetic (Scandicaine®) was injected subcutaneously, and a longitudinal 2 cm skin incision was made on the dorsum of the foot. The lymphatic vessels were carefully dissected free. Using a special 22G spring-loaded needle (Unimed S.A.) with a stylet, the most visible lymphatic vessel was cannulated. Adhesive stripes were used to anchor the needle and infusion line. Using a syringe pump with an injection speed of 6–8 ml/h, Lipiodol was injected at a rate of up to 1 ml/10 kg body weight per foot, up to a maximum volume of 20 ml of iodised oil (48% iodised glycerol ester, Lipiodol® Ultra-Fluid, Guerbet GmbH, Sulzbach, Germany). To monitor the flow of Lipiodol through the lymphatic vessels and to rule out inadvertent venous injection, fluoroscopy was performed. As soon as a fistula was visible or 20 ml of Lipiodol had been injected, the materials were removed, and the incision was carefully disinfected and sutured. The sutures could be removed 10 to 12 days after the procedure. At the end of the procedure, a fluoroscopy of the hip and groin region is usually performed at the surgical site (Fig. 4). The lymphatic fistula was visualised within 24 h of lymphangiography using X-ray or CT imaging to monitor lymph drainage and rule out further complications (Fig. 5). The only complication of this technique was when it was not possible to puncture the lymphatic vessel, as it was too fragile. If the lymphatic fistula did not close after lymphangiography, either surgical intervention or radiation therapy was performed.

### Technical success

The technical success of conventional lymphangiography was defined by the effective administration of contrast agents, aimed at providing a clear visualisation of the lymphatic system and all associated lymphatic abnormalities using X-ray images.

### Clinical outcome assessment

Therapeutic success was defined as the complete closure of the inguinal lymph fistula following lymphangiography, without the necessity for further surgical or radiotherapeutic interventions. Furthermore, the interval between the day of lymphangiography and the day of fistula closure was recorded, along with whether X-ray or CT imaging was performed after lymphangiography, as well as whether surgical intervention or radiation therapy was required in cases of persistent lymphatic fistula. These data were documented in an Excel (2011) spreadsheet.

Safety was evaluated based on complications following lymphangiography, including but not limited to contrast leakage, wound infection, allergic reactions, embolic events, or other adverse events. The Wilcoxon–Mann–Whitney *U* test was used to measure the correlation between the amount of lymphatic leakage and therapeutic success, as well as between the injected volume of Lipiodol and therapeutic success. The statistical analysis was performed using the software BiAS version 11.12 (epsilon-Verlag GbR, Frankfurt am Main, Germany). Additionally, a logistic regression test was carried out using JASP software to determine whether therapeutic success was influenced by the amount of injected Lipiodol or the volume of lymphatic drainage. A *p*-value of ≤ 0.05 was deemed statistically significant in both analyses.

## Results

### Patient clinical information

Conventional lymphangiography was performed in all 35 patients. Before the procedure, the average daily volume of lymphatic drainage was 217.43 ml/day. The mean median value was 200 ml/day. Drainage volumes varied considerably, ranging from 50 to 500 ml/day. The amount of Lipiodol administered varied between 4 and 18 ml, averaging 8.77 ml. The median value was 9 ml.

### Clinical outcomes

**Fig. 6:**
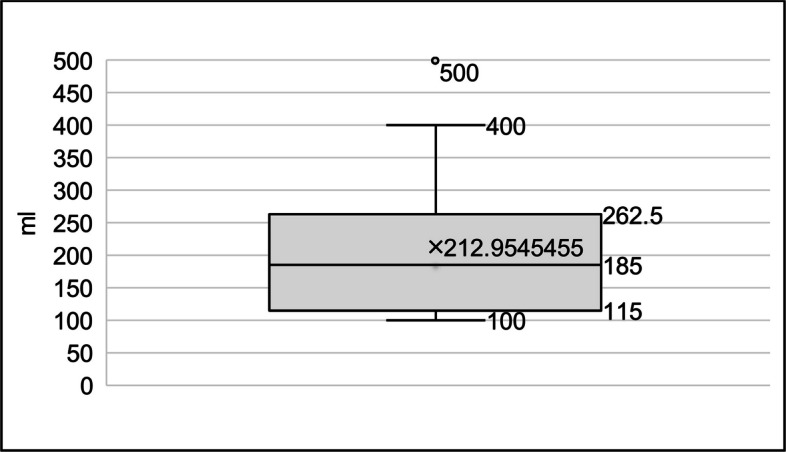
Daily drainage output in clinically successful patients prior to lymphangiography. Box plot depicting daily lymph fistula drainage volumes in 22 clinically successful patients prior to lymphangiography. The mean drainage volume was 212.95 millilitres per day (ml/day) (indicated by the “ × ” symbol), with a median value of 185 ml/day. The interquartile range extended from 115 to 262.5 ml/day, and the overall range was 100 to 500 ml/day

In a cohort of 35 patients, the fistula site was identified and radiologically documented, achieving a technical success rate of 100%. In 22 cases, corresponding to a therapeutic success rate of 62.86%, complete closure of the lymphatic fistula was accomplished following conventional lymphangiography, eliminating the need for further surgical or radiotherapeutic interventions. The 22 clinically successful patients exhibited an average daily lymph fistula volume of 212.95 ml/day prior to the procedure (Fig. 6). An average of 9 ml of Lipiodol was used, with amounts ranging from 4 to 18 ml. Only in these 22 patients was it feasible to evaluate the interval between the day of lymphangiography and the day the lymph fistula closed. This interval ranged from 1 to 35 days, with an average closure time of 7.13 days. The follow-up period was restricted to the duration until the lymphatic fistula closed after lymphography or until the patient required additional intervention in the event that lymphography alone was ineffective. Most of these patients (17) had malignant melanoma as their underlying disease. All clinically successful patients underwent an X-ray the day after lymphangiography, except for one patient who had a CT scan instead. No complications occurred in any of these 22 patients.

Based on the lymphographic findings, three of the remaining 13 patients underwent surgery, specifically negative-pressure wound therapy, commonly referred to as vacuum therapy. The other six patients (17.14%) received radiation therapy to completely close the lymph fistula. Sufficient information on the subsequent course of the remaining four patients (11.43%) was not available. Among the three patients who underwent vacuum therapy, the mean age was 60 years (range, 24–79), and the mean lymphatic fistula volume was 250 ml (range, 50–500 ml). Their diagnoses included malignant melanoma, Merkel cell carcinoma, and macrophage activation syndrome, respectively. The average volume of Lipiodol used was 7.67 ml, with a range of 5 to 9 ml. The six patients who received radiotherapy had a mean age of 73.3 years (range, 51–81 years) and a mean lymphatic fistula volume of 279.16 ml (range, 150–500 ml). Each of these patients had malignant melanoma. On average, 9.16 ml of Lipiodol was used, with a range of 8 to 12 ml. The radiation dose consisted of five fractions of 2.4 Gy each over the course of a week, totalling a maximum dose of 48 Gy. Of the remaining four patients for whom follow-up data were lacking, one was diagnosed with malignant melanoma, while the others had Merkel cell carcinoma. Their mean age was 64.5 years (range, 43–78), and the average lymphatic fistula volume was 125 ml (range, 50–250 ml). The mean volume of Lipiodol administered was 7.75 ml, with a range of 6 to 9 ml.

The success of the therapy was independent of both the amount of lymphatic drainage and the amount of Lipiodol administered. The Wilcoxon–Mann–Whitney *U* test, employed for statistical analysis, indicated no significant difference in the distribution of the amount of Lipiodol administered and the therapeutic effect of lymphangiography (*p* = 0.51). Similarly, the daily amount of lymphatic drainage had no significant impact on the success rate of the therapy (*p* = 0.82). The logistic regression analysis also revealed no significant association between the volume of Lipiodol administered and the success of lymphangiography. The estimated coefficient for Lipiodol volume was positive (*β* = 0.108; *p* = 0.48), suggesting a tendency towards higher odds of treatment success with increasing volume. The corresponding odds ratio is approximately 1.11 per ml, indicating an increase in success odds of about 11% for each additional ml. However, this effect was not statistically significant. Likewise, no significant influence on treatment success was found for the daily lymphatic drainage volume. The estimated coefficient was slightly negative (*β* = − 0.001; *p* = 0.79), suggesting that higher drainage volumes might tend to be associated with lower success odds. The odds ratio here is close to 1 (≈ 0.999 per ml), indicating a negligible effect. This relationship was also not statistically significant.

## Discussion

Conventional lymphangiography was found to be safe and, in most cases, therapeutically effective. The current literature primarily comprises studies [8–13], which focus on different lymphatic flow locations, such as chylothorax, chylous ascites, thoracic and inguinal lymphatic fistulas, pelvic lymphatic fistulas, abdominal and peripheral lymphatic fistulas, thoracic, abdominal and peripheral lymphoceles, or cervical lymphoceles. In most of these studies, the total number of patients is 22 or fewer [10–12], with only three studies reporting larger populations of 355 [13], 64 [9], and 43 patients [8], respectively. The technical success rate lymphangiography—defined in a manner consistent with our study as the successful injection of Lipiodol into lymphatic vessels with the aim of clearly visualising the lymphatic system and all associated lymphatic abnormalities using fluoroscopy—varies in other studies from 86 to 100% [4, 8–13]. In instances where lymphangiography failed and where the fistula site could not be identified and radiologically confirmed, the reasons were mainly fragile lymphatic vessels, disturbances in contrast medium outflow, or lymphoedema [11, 34]. Meanwhile, the therapeutic success rate ranges between 51 and 75% and is similarly defined by the authors as the complete closure of the lymph fistula after lymphangiography without the need for further surgical or radiotherapeutic interventions [8–13]. In this study was evaluated the therapeutic value of conventional lymphangiography on a homogeneous patient group with exclusively persistent inguinal lymph fistulas after lymphadenectomy. Despite the differences in patient demographics compared to the abovementioned studies, our findings demonstrated a technical success rate of 100% and a therapeutic success rate of 62.86%, which align with earlier results. That contributes to supporting the notion that conventional lymphangiography may be effective in sealing persistent inguinal lymphatic fistulas after lymphadenectomy, in addition to serving as a reliable diagnostic tool. Additionally, in both our work and the studies listed above, there were no complications (including, but not limited to, contrast leakage, wound infection, allergic reactions, embolic events or other adverse events), demonstrating the safety of this treatment. Notably, the total time from the day of lymphangiography to the day of fistula closure varied from approximately 1 day to 4 weeks, correlating well with our findings of 1 day to 5 weeks. Furthermore, the median value (7.13 days) is nearly identical to the values reported by Kortes et al. [10] and Pan et al. [13] (5 days; other authors did not report the median). Additional therapies employed in the other studies for clinically unsuccessful patients included surgical interventions, immediate or delayed sclerotherapy, pleurosclerosis, and peritoneovenous shunt. In contrast, in our study almost half of the unsuccessful patients received radiotherapy (6 out of 13) and another third (4 out of 13) underwent surgery, lymphangiography was helpful in the planning of surgery due to the visualisation of the lymphatic defect.

The Wilcoxon–Mann–Whitney *U* test in our study indicated that there is no correlation between the injected lipiodol and therapeutic success (*p* = 0.51 and *p* = 0.49, respectively). Gruber-Rouh et al. reached the same conclusion [9], while other studies did not investigate such a correlation. However, Pan et al. [13] found that a significantly higher volume of Lipiodol was injected in patients with chylothorax than in those with inguinal lymph fistula (*p* = 0.007). Therefore, the patient population could be one reason for the absence of such a correlation. The results regarding the correlation between daily lymphatic drainage volume and therapeutic success rate are contradictory. In the study by Gruber-Rouh et al. [9], considering patients with a lymphatic drainage volume of less than 200 ml/day (instead of the original range of 10 to 1000 ml/day) would impact the success rate, which would be 96.8% instead of 70.3%. But even in patients with more than 200 ml/day, the therapeutic success rate was 58.1%. The same result regarding the correlation between drainage volume and therapeutic success came from Alejandre-Lafont et al. [8] where occlusion occurred in 70% of patients with a lymphatic drainage volume of less than 500 ml/day, while the success rate dropped to 35% with a lymphatic drainage volume of more than 500 ml/day. In contrast, in the study by Kortes et al. [10], occlusion was only observed in patients whose outflow volume exceeded 1000 ml/day. Pan et al. [13] report that a drainage volume greater than 500 ml/day correlates with treatment failure (*p* = 0.025). However, this correlation is based on a total of 258 patients (258 of 355 were treated with lymphangiography only), and they had various clinical diagnoses of lymphatic leaks. Out of the total cohort, only 134 patients had inguinal fistulas, with their drainage volume ranging from 200 to 400 ml per day and averaging 300 ml per day; thus, the general statement about the overall patient population does not apply exclusively to those with inguinal lymphatic fistulas. Our patients fall within almost the same drainage volume range (50 to 500 ml) and have an average of 217.43 ml/day. Most importantly, the average volume of lymph leakage in clinically successful patients was 212.95 ml/day, while for the 13 clinically unsuccessful patients, it was 225 ml/day. These findings suggest that the baseline severity of the lymph leak, as measured by the daily fistula volume, is not always a reliable predictor of therapeutic success in patients with drainage volumes of less than 500 ml/day. This implies that the therapeutic effect may be independent of the volume of lymphatic drainage present at the time of intervention.

The specific patient group contributes to this observation, as our study focused exclusively on patients with inguinal fistulas. This lack of correlation may indicate that other factors, such as the precise anatomical features of the leak or the underlying disease process, could have a more decisive influence on the effectiveness of lymphangiography. Despite the absence of a significant relationship between lymphatic drainage volume and therapeutic success (*p* = 0.82), our statistical analysis revealed a success rate of 62.86%, which is nearly identical to that reported for patients with inguinal lymphatic fistulas in the study by Pan et al. (62.2%) [13] and for various patients in the study by Alejandre-Lafont et al. (70%) [8]. Furthermore, it is noted that if the daily fistula volume in our study had exceeded 500 ml/day, this might have led to the correlation proposed by Alejandre-Lafont et al. [8].

The limitations of the present study include the absence of a prospective follow-up study, as retrospective analyses have such constraints as long-term outcomes after discharge or later complications are not always fully recorded. Additionally, the present study focuses solely on transpedal lymphangiography and not intranodal method. Lastly, there is insufficient data regarding other variables in clinically unsuccessful patients, such as the time interval between additional intervention and complete fistula closure and specific complications after radiotherapy or surgical intervention in this context.

## Conclusions

In conclusion, conventional lipiodol-based lymphangiography is effective and safe as an adjunct to conservative treatments. The lack of association between Lipiodol, or drainage volume, and outcome suggests that therapeutic success may depend more on anatomical and disease-specific factors, which underscores the necessity for a more individualised approach when selecting patients and planning subsequent interventions.

## Data Availability

All data generated or analysed during this study are included in this published article.

## Abbreviations

m: Male
f: Female
CT: Computed tomography
n: Number
p: Probability value
